# Lung cancer diagnosed on pap smear: A case report and review of the literature

**DOI:** 10.1016/j.gore.2021.100776

**Published:** 2021-05-07

**Authors:** Marie-Claire Leaf, Diana Pearre, Fabio Cappuccini, Krishnansu Tewari

**Affiliations:** a Department of Obstetrics and Gynecology, University of California, Irvine-Medical Center, Orange, CA, USA; b Division of Gynecology Oncology, Department of Obstetrics and Gynecology, University of California, Irvine-Medical Center, Orange, CA, USA

**Keywords:** Lung cancer, Pap smear

## Abstract

• Pap smear test can detect metastases of extragenital malignancies.
• Metastases of extragenital cancers to the cervix are predominantly adenocarcinomas.
• Immunostaining is critical in determining the primary cancer site.

## 1 Introduction

The pap smear has been used for 70 years in the screening of cervical cancer and is considered one of the most successful public health measures to prevent cervical cancer. Since its invention, cytologic techniques have improved with the creation of liquid-based cytology (Shaw, 2000). Cytology is now being used in the detection of lung cancer from sputum specimens, bronchoalveolar lavage (BAL), bronchial washings (BWs), and bronchial brushings (Fan et al., 2010). When biopsies cannot be obtained, conventional cytology is used for the detection of lung cancers, but there is evidence that liquid-based cytology may have significantly higher diagnostic sensitivity for lung cancer than conventional pap smear method (Fan et al., 2010).

This is the first reported case of lung adenocarcinoma diagnosed on a pap smear from cervical cells. Lung cancers most commonly metastasize to the brain, bones, and liver (Myers and Wallen, 2020), however this case describes the unusual metastatic location of a lung cancer to the uterine cervix, diagnosed on a cervical cancer-screening test. To date, there are only six reported cases of lung cancers with metastases to the cervix.

## 2 Case

A 53 year-old woman presented for three-month onset of abdominal bloating and difficulty voiding. On review of system, the patient admitted to dyspnea on exertion. She did not have any past medical or surgical history. Her social history was significant for a 30 pack-year tobacco use. Physical examination was within normal limits with clear lungs on auscultation and normal pelvic examination. A pap smear was performed and showed atypical glandular cells (AGC), negative for high-risk human papilloma virus (HPV). A transvaginal ultrasound demonstrated a normal size uterus with an endometrial stripe of 0.3 centimeter (cm). The patient subsequently underwent a colposcopy. A small lesion was noted on the exocervix at 11o’clock and a biopsy was performed, along with an endocervical curettage (ECC). The biopsy was non-diagnostic with benign squamous mucosa, however the ECC revealed focal atypical cells, suspicious for malignancy.

**Fig. 1:**
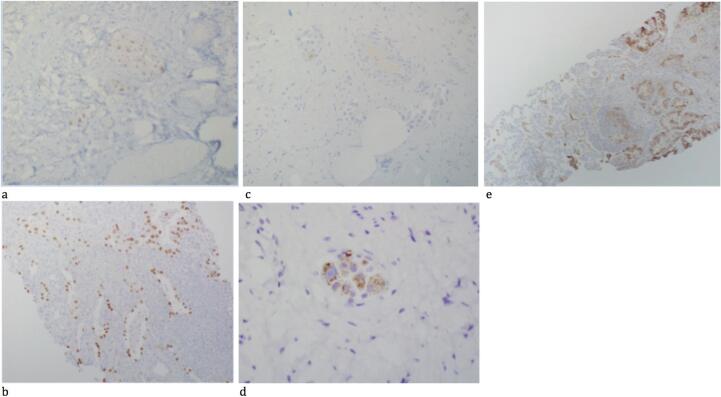
(a) Cervical biopsy TTF-1 positive. (b) Lung biopsy TTF-1 positive. (c) Cervical biopsy p16 negative. (d) Cervical biopsy Napsin-A positive. (e) Lung biopsy PDL-1 positive.

**Fig. 2:**
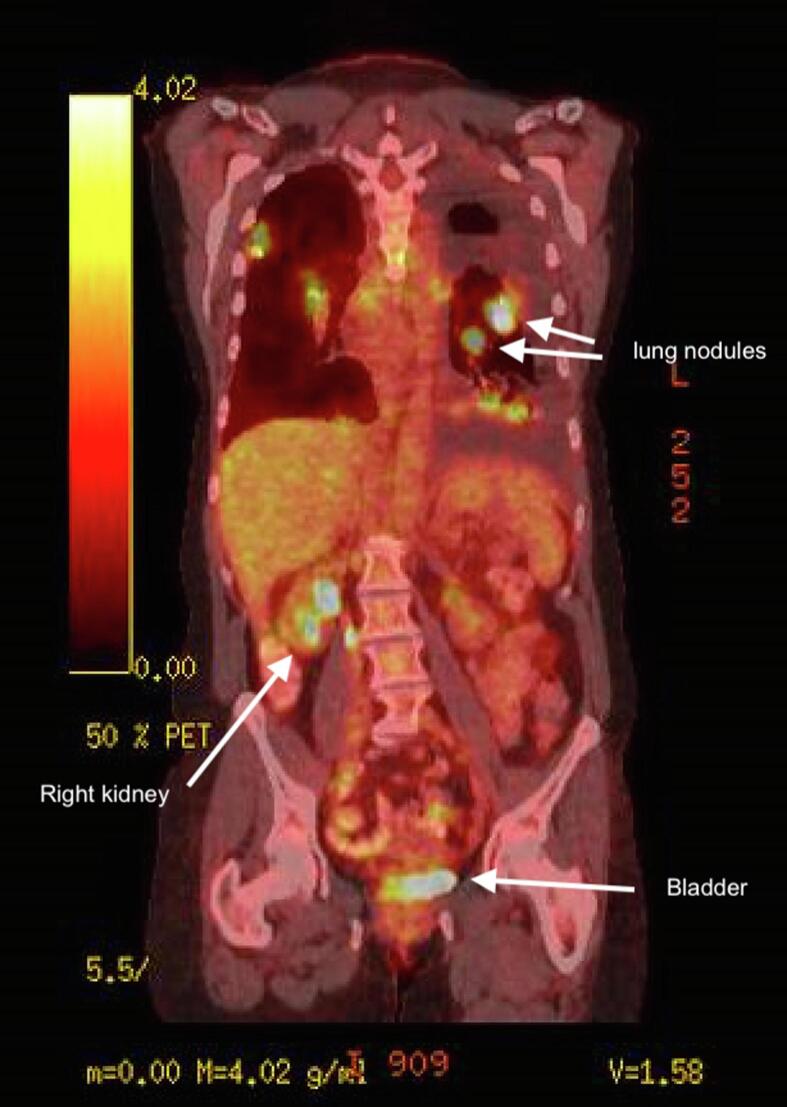
PET/CT scan demonstrating lung nodules and absence of abnormal abdominal and pelvic findings.

The patient was referred to gynecologic oncology and she underwent a loop electrosurgical excision procedure (LEEP) and dilation and curettage (D&C). Pathology resulted with benign endometrial epithelium, mild focal squamous atypia, and the cervical excision revealed malignant cells present within the lymphatics, negative for cyclin-dependent kinase inhibitor 2A (p16), typically positive in cervical cancers. The sample was positive for Napsin-A and thyroid transcription factor (TTF-1), concerning for lung adenocarcinoma (Fig. 1). She underwent a Positron Emission Tomorgraphy/Computerized Tomography (PET/CT) scan demonstrating multiple pulmonary nodules, the largest measuring 3 cm. There were multiple subcentimeter mediastinal lymph nodes and a 2.7 cm hypermetabolic left paratracheal lymph node. Moderate bilateral pleural effusions were noted with no abnormal findings in the abdomen or pelvis (Fig. 2).

Ultimately, a CT-guided biopsy of the lung nodule was performed and confirmed moderately differentiated adenocarcinoma originating from the lung. The endocervical biopsies were compared to the lung biopsy and had similar histology. She was subsequently referred to hematologic oncology and started on Pembrolizumab as the tumor was found to have high expression of Programmed Death-ligand 1 (PD-L1) (90%) and she declined chemotherapy. Six months later, she was found to be progressing, and is currently on standard therapy with Carboplatin, Pemetrexed, and Pembrolizumab.

## 3 Discussion

To our knowledge, this is the first reported case of a cervical biopsy that led to the diagnosis of primary lung carcinoma. Although the cervix appeared normal, malignant cells were identified on biopsy, raising suspicion for a primary cervical cancer. Diagnosis was eventually made with additional immunostaining confirming this as a malignancy originating from the lung.

Metastases of extragenital malignancies to the cervix are rare and have mostly been described from the gastrointestinal tract or breast (Mazur et al., 1984). On a detailed review of Pubmed, there were six cases identified with the spread of lung cancer to the cervix (Mazur et al., 1984, Hollier et al., 1997, Khan et al., 2007, Kai et al., 2009, Chuang et al., 2018, Yan et al., 2019) (Table 1). Of the six described cases, all were adenocarcinomas. Three of the six cases were recurrent disease to the cervix. Other cases prompted cervical biopsies secondary to visible lesions. Thus, this is one of very few cases that describes the detection of a primary lung cancer presenting with cervical metastasis.

**Table 1:** Lung cancer metastatic to the uterine cervix. CK = cytokeratin; TTF = thyroid transcription factor, PET = Positron Emission Tomography, GATA = GATA binding protein, CA 125 = Cancer antigen 125, WT1 = Wilms’ tumor 1.

Case	Smoker	Lung cancer type	Prior diagnosis of lung cancer	Metastatic sites	Hysterectomy performed	Pelvic/Cervical exam	Immunostaining
Mazur et al. (1984)	Unknown	Adeno-carcinoma	Yes	Cervix, ovaries (recurrence)	Yes	“Rapidly growing nodular lesions in the cervix”	Not described
Hollier et al. (1997)	Unknown	Adeno-carcinoma	Yes	Cervix (recurrence)	Yes Free of disease for 7 months post-surgery	“Visible cervical lesion”	Not described
Khan et al. (2006)	Unknown	Adeno-carcinoma	No	Not described	No Initiated medical management	“Thickening of the external os at 6o’clock, otherwise normal”	**TTF-1 (+)** Thyroglobulin (-)
Kai et al. (2009)	Unknown	Adeno-carcinoma	Yes	Cervix (recurrence)	No Initiated medical management for recurrence. Death 12 months later.	“Cauliflower type tumor in the uterine cervix”	**TTF-1 (+)** Thyroglobulin (-)Pulmonary surfactant apoprotein A (+)CA125 (-)
Chuang et al. (2018)	Nonsmoker	Adeno-carcinoma	No	Breast, cervix	No Initiated medical management	“Transvaginal ultrasound suggestive of cervical carcinoma”	**TTF-1(+)** CK-7(+) Ki-67 (25%) GATA-3(-) **P16(-)** P63(-)
Yan et al. (2019)	Nonsmoker	Adeno-carcinoma	No	Cervix	No Initiated medical management	Not described – biopsy performed given hypermetabolic activity at the cervix on PET scan.	**TTF-1(+)** CK-7(+) CK-20(-)
Current case	Smoker	Adeno-carcinoma	No	Paratracheal lymph nodes, mediastinal lymph nodes	No Initiated medical management	Normal size uterus with an endometrial stripe of 0.3 cm. Small lesion at 11o’clock of the exocervix which was benign	**TTF-1 (+)** **P16 (-)** WT1 (-) CK-7 (+)

Although cervical cancer metastasizes to the lung much more frequently than lung cancer to the cervix (with a frequency of 4.1–7.7%) (Li et al., 2016), appropriate immunostaining facilitated the diagnosis of metastatic pulmonary malignancy. Cervical cancers are often p16 positive (Sun et al., 2019) while lung adenocarcinoma are Napsin A and TTF-1 positive (Inamura, 2018). PD-L1 (CD274) may be present in both lung and cervical malignancies (Inamura, 2018). Thus, a cervical biopsy negative for p16 should prompt additional immunostaining for extragenital adenocarcinomas such as breast, lung, and colon, the three most prevalent cancers in women (Siegel et al., 2021). This case stresses the importance of immunostaining in identifying the primary source of malignancy.

There is paucity of data regarding lung cancer metastasis to the cervix, however caution must be taken when a pap smear results with adenocarcinoma and a pelvic exam is not consistent with cervical cancer or in patients with known extragenital primary adenocarcinoma. Immunostaining is critical in determining the primary cancer site as the misdiagnosis of extragenital metastasis to the cervix could lead to a delay in the correct treatment.

## Author contribution

Primarily Marie-Claire Leaf wrote the manuscript with contribution from Diana Pearre. Fabio Cappuccini and Krishnansu Tewari were the final editors of the manuscript and oversaw the project.

## Declaration of Competing Interest

The authors declare that they have no known competing financial interests or personal relationships that could have appeared to influence the work reported in this paper.
